# An Excessive Dose of Sulphonal

**Published:** 1900-01-27

**Authors:** 


### AN EXCESSIVE DOSE OF SULPHONAL.

DR. PERCY SMITH,[1] late resident physician Bethlem

Royal Hospital, records a case in which a patient took two doses of sulphonal of 150 grains each, apparently within the course of about twenty-four hours. The patient was a lady suffering from melancholy, who for no particular object, unless it might be to procure sleep, took these enormous doses, which she had obtained quite in an ordinary way from a chemist. The tale has two morals; one, perhaps, is that an excessive dose of sulphonal is not quite so dangerous as some people have imagined, the toxic effects usually arising slowly from taking repeated doses for a long time rather than from a single one, even though excessive; the other that it is perfectly possible for patients to get from chemists what drugs they like, and practically in what quantities they like, however potent they may be, so that they are not on the schedule. Dr. Percy Smith remarks on the fact that the drug was not obtained in the form of a proprietary article, but of a standard B.P. preparation, and was dispensed without prescription, and on making inquiries at the chemists where the drug was obtained he was assured that there was no difficulty about the matter, and that no suspicion would be excited by a person asking for six powders with 25 grains of sulphonal in each.

